# Prognostic value of estimated glomerular filtration rate in hospitalised older patients (over 65) with COVID-19: a multicentre, European, observational cohort study

**DOI:** 10.1186/s12877-022-02782-5

**Published:** 2022-02-12

**Authors:** Ben Carter, Euan A. Ramsay, Roxanna Short, Sarah Goodison, Jane Lumsden, Amarah Khan, Philip Braude, Arturo Vilches-Moraga, Terence J. Quinn, Kathryn McCarthy, Jonathan Hewitt, Phyo K. Myint, On Behalf of COPE Study, Eilidh Bruce, Alice Einarsson, Kirsty McCrorie, Ken Aggrey, Jimmy Bilan, Kerr Hartrop, Caitlin Murphy, Aine McGovern, Enrico Clini, Giovanni Guaraldi, Alessia Verduri, Carly Bisset, Ross Alexander, Joanna Kelly, Caroline Murphy, Tarik El Jichi Mutasem, Sandeep Singh, Dolcie Paxton, Will Harris, Susan Moug, Norman Galbraith, Emma Bhatti, Jenny Edwards, Siobhan Duffy, Maria Fernanda Ramon Espinoza, Thomas Kneen, Anna Dafnis, Hala Allafi, Maria Narro Vidal, Angeline Price, Lyndsay Pearce, Thomas Lee, Shefali Sangani, Madeline Garcia, Charlotte Davey, Sheila Jones, Kiah Lunstone, Alice Cavenagh, Charlotte Silver, Thomas Telford, Rebecca Simmons

**Affiliations:** 1 Biostatistics and Health Informatics, Institute of Psychiatry, Psychology & Neuroscience, King’s College London, De Crespigny Park, London, SE5 8AF UK; 2 Ageing Clinical & Experimental Research Team, Institute of Applied Health Sciences, University of Aberdeen, Aberdeen, Scotland AB25 2ZD; 3 Forensic and Neurodevelopmental Sciences, Institute of Psychiatry, Psychology & Neuroscience, King’s College London, De Crespigny Park, London, SE5 8AF UK; 4 Department of Geriatric Medicine; Ysbyty Ystrad Fawr, Aneurin Bevan University Health Board, Hengoed, Wales CF82 7GP; 5 Department of Geriatric Medicine, Glasgow Royal Infirmary, NHS Greater Glasgow & Clyde, G4 0SF, Glasgow, UK; 6 Ageing and Complex Medicine Department, Salford Royal NHS Foundation Trust, Stott Lane, Manchester, M6HD8 UK; 7 Department of Surgery and Care of the Elderly, Southmead Hospital, North Bristol NHS Trust, Bristol, BS10 5NB UK; 8 Institute of Cardiovascular and Medical Sciences, University of Glasgow, Glasgow, G12 8TA UK; 9 Division of Population Medicine, Cardiff University, Cardiff, Cf10 3XQ UK

**Keywords:** COVID-19, Chronic kidney failure, eGFR, Mortality, Dose-response

## Abstract

**Background:**

The reduced renal function has prognostic significance in COVID-19 and it has been linked to mortality in the general population. Reduced renal function is prevalent in older age and thus we set out to better understand its effect on mortality.

**Methods:**

Patient clinical and demographic data was taken from the COVID-19 in Older People (COPE) study during two periods (February–June 2020 and October 2020–March 2021, respectively). Kidney function on admission was measured using estimated glomerular filtration rate (eGFR). The primary outcomes were time to mortality and 28-day mortality. Secondary outcome was length of hospital stay. Data were analysed with multilevel Cox proportional hazards regression, and multilevel logistic regression and adjusted for individual patient clinical and demographic characteristics.

**Results:**

One thousand eight hundred two patients (55.0% male; median [IQR] 80 [73–86] years) were included in the study. 28-day mortality was 42.3% (*n* = 742). 48% (*n* = 801) had evidence of renal impairment on admission. Using a time-to-event analysis, reduced renal function was associated with increased in-hospital mortality (compared to eGFR ≥ 60 [Stage 1&2]): eGFR 45–59 [Stage 3a] aHR = 1.26 (95%CI 1.02–1.55); eGFR 30–44 [Stage 3b] aHR = 1.41 (95%CI 1.14–1.73); eGFR 1–29 [Stage 4&5] aHR = 1.42 (95%CI 1.13–1.80). In the co-primary outcome of 28-day mortality, mortality was associated with: Stage 3a adjusted odds ratio (aOR) = 1.18 (95%CI 0.88–1.58), Stage 3b aOR = 1.40 (95%CI 1.03–1.89); and Stage 4&5 aOR = 1.65 (95%CI 1.16–2.35).

**Conclusion:**

eGFR on admission is a good independent predictor of mortality in hospitalised older patients with COVID-19 population. We found evidence of a dose-response between reduced renal function and increased mortality.

**Supplementary Information:**

The online version contains supplementary material available at 10.1186/s12877-022-02782-5.

## Introduction

Initially, COVID-19 was described as primarily respiratory in nature and involvement of kidneys was not widely reported [1, 2]. However, further literature has described the increased presence of worsening kidney function concurrent with COVID-19 infection [3]. A number of potential mechanisms of kidney injury have been described, including direct viral infection of the kidneys, leading to acute tubular injury and endothelial damage as well as mechanisms secondary to systemic illness including sepsis and hypovolaemia [4, 5].

Estimated glomerular filtration rate (eGFR), is a calculation based on serum creatinine, age, race, sex and body size, and is used clinically as a measure of kidney function [6]. It is well described as a good indicator of mortality in non-COVID-19 patients with both acute and chronic kidney disease (CKD) [7] and in COVID-19 patients [8]. Lower baseline eGFR has also been shown to lead to increased rates of acute kidney injury (AKI), and renal replacement therapy in COVID-19 patients [9]. Lower eGFR was also more commonly seen in multimorbid patients and older people [10]. Several studies have reported a decline in renal function as a binary threshold of eGFR is associated with increased mortality in COVID-19 patients [10, 11] but few have reported on the association between the increasing severity of categorised eGFR and mortality in COVID-19 [12, 13].

Previous studies in older adults (> 65 years) showed that prevalence of CKD on admission with COVID-19 was 11.4% [14], and development of AKI ranged between 24.8% [14] and 39% [14]. Indeed, older age and eGFR (less than 60) [12] have been well described as a risk factors for mortality [15–17] alongside AKI development [18] and subsequent mortality in COVID-19 [19]. In addition, older age is associated with increased serum creatinine levels on admission in COVID-19 patients [20]. To date only the Geriatric Medicine Research Collaborative [13] and Xu et al. [21] have explored the gradated relationship between eGFR decline and mortality in older adults with COVID-19.

The aim of this paper is to determine the relationship between eGFR on admission to hospital with COVID-19 infection and clinical outcomes including mortality, and length of stay in older adults, using data from the COVID-19 in Older People (COPE) Study [16].

## Methods

### Study population and setup

This study was an extension of the COVID-19 in Older People (COPE) Study, with additional participants included in the second wave. The primary COPE study was a multicentre, observational study with 13 centres in both the UK and Italy [16]. The study protocol for the original COPE study has previously been published [22], with this study following the same study design. Approval for the study was granted in the UK by the Health Research Authority (20/HRA/1898) and in Italy from the Ethics Committee of Hospital Policlinico Modena (Reference 369/2020/OSS/AOUMO). Data was collected using a standardised case report based on hospital records and entered into a centrally co-ordinated InferMed MACRO database housed within King’s College London. Data protection policies were adhered to at each hospital.

### Participants

Consecutive patients aged 65 years or older, who were admitted to hospital at any of the recruiting centres during the first wave (27th February to 10th June 2020) and the second wave (1st October 2020 – 8th March 2021) with a COVID-19 diagnosis were included in the present study. Patients aged 65 years or older who developed COVID-19 whilst already hospitalised for a different reason (nosocomial infections) were also included. Nosocomial infection was assumed if the date of diagnosis was more than 5 days after the date of admission [23]. Diagnostic criteria for COVID-19 included a laboratory confirmed positive swab for SARS-CoV-2, and a clinical diagnosis based on signs, symptoms and radiological reporting consistent with COVID-19. There were no exclusion criteria applied.

### Outcomes

The primary outcome was mortality (time-to-mortality and 28-day mortality). The secondary outcome was length of stay in hospital (time from admission, or from diagnosis for nosocomial cases to discharge). Patients who were discharged prior to day-28 were imputed as having survived at day 28. Patients who died were censored in the time-to-discharge analysis.

### Primary exposure

Renal function was the primary exposure under investigation assessed as eGFR (CKD-EPI) on admission and was categorised into: Stage 1&2 (normal kidney function to mild loss of kidney function) (eGFR ≥ 60 ml/min/1.73m^2^); Stage 3a (mild to moderate loss of kidney function, eGFR 45–59 ml/min/1.73m^2^); Stage 3b (moderate to severe loss of kidney function, eGFR 30–44 ml/min/1.73m^2^); Stages 4 and 5 (Severe to complete loss of kidney function, eGFR 1–29 ml/min/1.73m^2^) [24].

### Covariates

Clinical characteristics collected included: sex; age, smoking status (current smoker, previous smoker and never smoker); C-reactive protein (CRP) levels at admission [25] and a diagnosis of diabetes mellitus, hypertension or coronary artery disease (CAD) present at admission. Patient’s frailty was assessed in-hospital, based on frailty status 2 weeks prior to admission using the Clinical Frailty Scale (CFS) [26, 27].

### Terminally ill patients

Due to very few patients with a terminal illness (CFS 9) being included in the study, they were excluded from analyses.

### Statistical analysis

There were 55 patients with missing smoking data, who were recorded as ‘never smokers’, and a further 64 patients with a missing eGFR recording, inputted as having an eGFR ≥60. Clinical characteristics from both waves were compared by in hospital mortality. Time-to-event outcomes (mortality and time to discharge) were analysed with multilevel multivariable Cox proportional hazards (PH) regression models. Each Cox regression model fitted the hospital site as a random intercept effect, to account for heterogeneity across sites. Crude hazard ratios (HR) and adjusted hazard ratios (aHR) are presented alongside the associated 95% Confidence Intervals (CI). The PH assumption was assessed visually using log-log plots. Analyses were performed using Stata SE version 16 (StataCorp LLC; College Station, TX), Kaplan-Meier and subgroup forest plots were visualised in R.

28-day mortality was analysed with a multivariable multilevel logistic regression model, which fitted hospital site as a random effect, with crude Odds Ratios (OR) and adjusted Odds Ratios (aOR) and associated 95% CIs. All models were adjusted for: eGFR; wave 1 or wave 2; age (categorised into: 65–74 years, 75–84 years, 85-94 years, ≥ 95 years); sex (male or female); smoking status (never smoker, current smoker or previous smoker); diabetes (yes or no); hypertension (yes, yes and on treatment or no); coronary artery disease (yes or no); C-reactive protein on admission (0–39 mg/dl or ≥ 40 mg/dL [25]); Clinical Frailty Scale (categorised into: CFS 1–3, CFS 4, CFS 5–6, CFS 7–8, CFS 9).

### Dose-response

This was assessed in each analysis using a test for post estimation linear test for trend to the adjusted analyses and presented as the linear change from each category of renal failure compared to Stage1&2.

### Subgroup analyses

Subgroups analyses were carried out for each outcome using the multivariable multilevel analyses as above. These present associations of eGFR (categorised using the established binary cut off for reduced renal function: eGFR 1–59 ml/min/1.73m^2^, eGFR ≥ 60 ml/min/1.73m^2^) with mortality and time-to-discharge, within each subgroup.

## Results

**Fig. 1:**
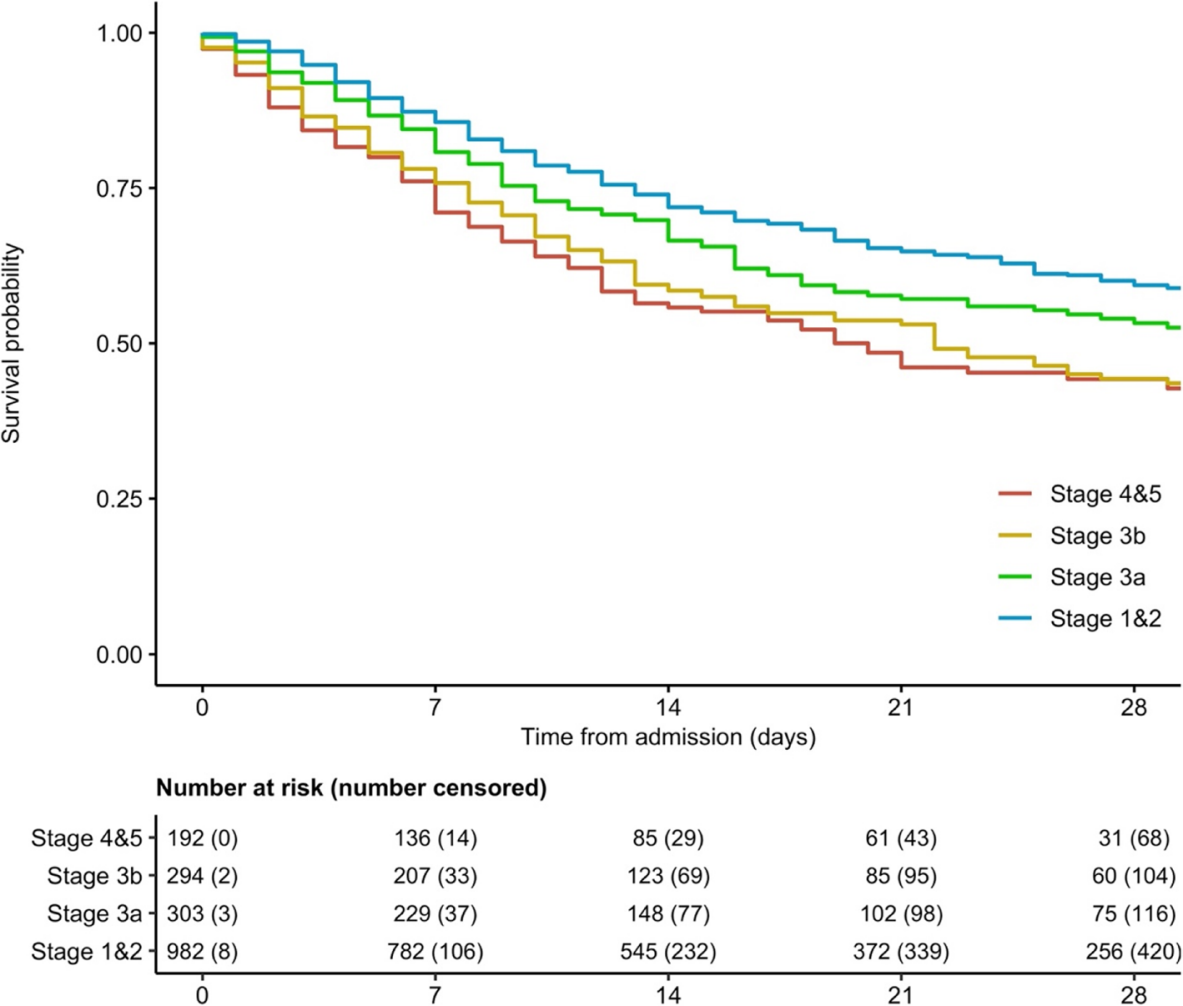
A Kaplan Meier survival function to assess categorised admission eGFR^&^ on the time to mortality for patients hospitalised with COVID-19

A total of 1802 patients aged ≥ 65 years were included in the study (Wave 1, *n* = 1318; Wave 2, *n* = 484). The mean age was 79.6 (range 65–101, SD 7.98), and 992 (55.0%) were male (Table 1). The median (IQR) time between admission and mortality was 14 days [7–27]. All cause in-hospital 28-day mortality was 42.3% (*n* = 742). 28-day mortality was higher in older age groups (56.5% in those aged 95+ years; 48.2% in those aged 85–94; 42.8% in those aged 75–84; 31.2% in those aged 65–74), in patients at increasing stages of renal failure on admission (53.5% at Stages 4 and 5; 48.3% at Stage 3b; 42.7% at Stage 3A; 36.0% at Stages 1 and 2, Fig. 1), and patients with co-morbidities including coronary artery disease (45.6% vs 39.6%) and diabetes (42.6% vs 40.6%), and in those with an increased frailty score (Table 1).

**Table 1:** Included Population description

	Alive (*N* = 1060)	Dead (*N* = 742)	Total (*N* = 1802)
	N (%)	N (%)	N (%)
eGFR			
1–29	92 (46.5)	106 (53.5)	198 (11.0)
30–44	153 (51.7)	143 (48.3)	296 (16.4)
45–59	176 (57.3)	131 (42.7)	307 (17.0)
60+	600 (64.0)	337 (36.0)	937 (52.0)
Missing	39	25	64
Wave			
1	765 (58.0)	553 (42.0)	1318 (73.1)
2	295 (61.0)	189 (39.0)	484 (26.9)
Age			
65–74	362 (68.8)	164 (31.2)	526 (29.2)
75–84	431 (57.2)	322 (42.8)	753 (41.8)
85–94	247 (51.8)	230 (48.2)	477 (26.5)
95+	20 (43.5)	26 (56.5)	46 (2.6)
Sex			
Female	509 (62.9)	300 (37.1)	809 (44.9)
Male	550 (55.4)	442 (44.6)	992 (55.0)
Missing	1	0	1
Smoking			
Never Smokers	515 (60.8)	332 (39.2)	847 (47.0)
Ex-smokers	444 (55.7)	353 (44.3)	797 (44.2)
Current Smokers	67 (65.0)	36 (35.0)	103 (5.7)
Missing	34	21	55
Diabetes			
No	764 (59.4)	522 (40.6)	1286 (71.4)
Yes	294 (57.4)	218 (42.6)	512 (28.4)
Missing	2	2	4
Hypertension			
No	452 (57.4)	335 (42.6)	787 (43.7)
Yes	181 (61.1)	115 (38.9)	296 (16.4)
Yes & on treatment	427 (59.4)	292 (40.6)	719 (39.9)
CAD			
No	807 (60.4)	528 (39.6)	1335 (74.1)
Yes	252 (54.4)	211 (45.6)	463 (25.7)
Missing	1	3	4
CRP			
0–40	401 (69.4)	177 (30.6)	578 (32.1)
> 40	659 (53.8)	565 (46.2)	1224 (67.9)
CFS			
CFS 1–3	283 (71.1)	115 (28.9)	398 (22.1)
CFS 4	157 (59.5)	107 (40.5)	264 (14.7)
CFS 5–6	368 (57.4)	273 (42.6)	641 (35.6)
CFS 7–8	227 (50.9)	219 (49.1)	446 (24.8)
CFS 9	9 (29.0)	22 (71.0)	31 (1.7)
Missing	16	6	22

### Primary outcomes: time to mortality and 28-day mortality

In the crude Cox proportional hazards regression, eGFR was associated with increased mortality (Table 2). In the multilevel multivariable Cox PH regression, reduced renal function was associated with increased mortality (compared to Stages 1 and 2): Stage 3a aHR = 1.26 (95%CI 1.02–1.55); Stage 3b aHR = 1.41 (95%CI 1.14–1.73); Stages 4 and 5 aHR = 1.42 (95%CI 1.13–1.80) (Table 2, Fig. 1). In addition to this increasing age, male sex, CRP ≥40 mg/dL, and a CFS score ≥ 5 were associated with increased mortality (Table 2). There was very clear evidence of a linear test for trend (aHR = − 0.13; 95%CI -0.21, − 0.05; *p* = 0.002).

**Table 2:** Crude and multivariable Cox proportional hazards regression, presenting the crude Hazard Ratio (HR) and adjusted HR (aHR) for the time to mortality

	Crude		Multivariable	
	HR (95%CI)	*p*	aHR	*p*
eGFR (60+)				
1–29	1.55 (1.24–1.95)	< 0.001	1.42 (1.13–1.80)	0.0031
30–44	1.54 (1.26–1.89)	< 0.001	1.41 (1.14–1.73)	0.0014
45–59	1.30 (1.06–1.60)	0.0125	1.26 (1.02–1.55)	0.0295
Wave 2	0.75 (0.62–0.91)	0.0036	0.77 (0.63–0.94)	0.0093
Age (65–74)				
75–84	1.52 (1.25–1.85)	< 0.001	1.44 (1.17–1.77)	< 0.001
85–94	1.78 (1.44–2.21)	< 0.001	1.60 (1.27–2.01)	< 0.001
95+	2.74 (1.78–4.22)	< 0.001	2.61 (1.67–4.09)	< 0.001
Male	1.13 (0.97–1.32)	0.1203	1.20 (1.02–1.41)	0.0279
Smoking (Never)				
Ex-smoker	1.22 (1.05–1.42)	0.0112	1.14 (0.97–1.33)	0.1204
Current smoker	0.96 (0.67–1.38)	0.8157	0.94 (0.65–1.37)	0.7450
CRP > 40	1.81 (1.52–2.16)	< 0.001	1.81 (1.51–2.16)	< 0.001
Diabetes	1.03 (0.88–1.22)	0.6902	0.99 (0.83–1.17)	0.8650
CAD	1.17 (0.99–1.38)	0.0722	1.03 (0.87–1.23)	0.7170
Hypertension (No)				
Yes	0.90 (0.72–1.12)	0.3506	0.91 (0.72–1.14)	0.4008
Yes & on treatment	0.86 (0.72–1.01)	0.0682	0.85 (0.71–1.01)	0.0653
CFS (1–3)				
CFS 4	1.39 (1.06–1.81)	0.0158	1.28 (0.97–1.67)	0.0791
CFS 5–6	1.48 (1.18–1.86)	< 0.001	1.30 (1.02–1.66)	0.0327
CFS 7–8	1.91 (1.50–2.43)	< 0.001	1.63 (1.26–2.11)	< 0.001

*Note*: aHR adjusted for eGFR, wave, age, sex, smoking status, CRP, diabetes, CAD, hypertension and CFS

For 28-day mortality, similar findings were reported with a clearer dose-response for worsening renal function linked to increased mortality (Table 3). The Stage 3a adjusted odds ratio (aOR) = 1.18 (95%CI 0.88–1.58); Stage 3b aOR = 1.40 (95%CI 1.03–1.89); Stages 4 and 5 aOR = 1.65 (95%CI 1.16–2.35). From the covariates increasing age, male sex, CRP > 40 mg/dl, and increasing frailty were associated with increased mortality in a multilevel logistic regression (Table 3). There was very clear evidence of a linear test for trend (aOR = − 0.19; 95%CI -0.31, − 0.06; *p* = 0.003).

**Table 3:** Multilevel logistic regression, presenting the crude Odds Ratio (OR) and adjusted OR (aOR) for 28-day mortality

	Crude		Multivariable	
	OR (95%CI)	*p*	aOR	*p*
eGFR (60+)				
1–29	1.97 (1.42–2.74)	< 0.001	1.65 (1.16–2.35)	0.0050
30–44	1.69 (1.28–2.23)	< 0.001	1.40 (1.03–1.89)	0.0303
45–59	1.24 (0.94–1.64)	0.1306	1.18 (0.88–1.58)	0.2647
Wave 2	0.71 (0.55–0.92)	0.0089	0.77 (0.59–1.01)	0.0634
Age (65–74)				
75–84	1.82 (1.41–2.35)	< 0.001	1.67 (1.27–2.19)	< 0.001
85–94	2.48 (1.86–3.31)	< 0.001	2.18 (1.58–3.00)	< 0.001
95+	5.94 (2.88–12.25)	< 0.001	4.58 (2.15–9.77)	< 0.001
Male	1.26 (1.03–1.55)	0.0269	1.48 (1.18–1.86)	< 0.001
Smoking (Never)				
Ex-smoker	1.12 (0.91–1.39)	0.2752	1.03 (0.82–1.30)	0.7844
Current smoker	0.92 (0.58–1.47)	0.7256	0.90 (0.54–1.49)	0.6794
CRP > 40	2.06 (1.64–2.59)	< 0.001	2.13 (1.67–2.71)	< 0.001
Diabetes	1.02 (0.81–1.27)	0.8849	0.98 (0.77–1.25)	0.9006
CAD	1.26 (1.00–1.58)	0.0494	1.07 (0.83–1.38)	0.5983
Hypertension (No)				
Yes	0.94 (0.70–1.27)	0.6904	0.92 (0.66–1.27)	0.6029
Yes & on treatment	0.82 (0.65–1.03)	0.0871	0.79 (0.62–1.01)	0.0584
CFS (1–3)				
CFS 4	1.68 (1.19–2.37)	0.0033	1.60 (1.12–2.30)	0.0104
CFS 5–6	2.01 (1.50–2.69)	< 0.001	1.75 (1.27–2.41)	< 0.001
CFS 7–8	2.79 (2.03–3.83)	< 0.001	2.36 (1.67–3.34)	< 0.001

*Note*: aHR adjusted for eGFR, wave, age, sex, smoking status, CRP, diabetes, CAD, hypertension and CFS

### Secondary outcome: time to discharge

There was no association, in either the crude or adjusted analysis, between stage of kidney disease and time to discharge. Compared to Stages 1 and 2, the adjusted analysis found a relationship between renal function and time to discharge: Stage 3a aHR = 1.09 (95%CI 0.89–1.32); Stage 3b aHR = 1.09 (95%CI 0.88–1.35); Stages 4 and 5 aHR = 0.84 (95%CI 0.64–1.09) (Table 4). There was no evidence for any dose-response (*p* = 0.21).

**Table 4:** Crude and multivariable Cox proportional hazards regression, presenting the crude Hazard Ratio (HR) and adjusted HR (aHR) for the time to discharge

	Crude		Multivariable	
	HR (95%CI)	*p*	aHR	*p*
eGFR (60+)				
1–29	0.79 (0.61–1.03)	0.0796	0.84 (0.64–1.09)	0.1833
30–44	0.97 (0.79–1.20)	0.8071	1.09 (0.88–1.35)	0.4255
45–59	0.99 (0.82–1.20)	0.9433	1.09 (0.89–1.32)	0.4121
Wave 2	0.61 (0.51–0.73)	< 0.001	0.60 (0.50–0.73)	< 0.001
Age (65–74)				
75–84	0.85 (0.72–0.99)	0.0417	0.89 (0.75–1.06)	0.1830
85–94	0.70 (0.58–0.85)	< 0.001	0.72 (0.58–0.89)	0.0022
95+	0.64 (0.33–1.25)	0.1935	0.77 (0.39–1.51)	0.4405
Male	0.97 (0.84–1.12)	0.7133	0.93 (0.80–1.08)	0.3394
Smoking (Never)				
Ex-smoker	1.07 (0.93–1.24)	0.3373	1.10 (0.94–1.28)	0.2235
Current smoker	0.98 (0.71–1.35)	0.9104	0.97 (0.70–1.35)	0.8677
CRP > 40	1.15 (0.99–1.33)	0.0712	1.07 (0.92–1.24)	0.4077
Diabetes	0.91 (0.78–1.06)	0.2364	0.92 (0.78–1.09)	0.3529
CAD	0.96 (0.82–1.13)	0.6435	1.01 (0.85–1.20)	0.9002
Hypertension (No)				
Yes	0.87 (0.71–1.08)	0.2059	0.97 (0.78–1.20)	0.7580
Yes & on treatment	1.09 (0.93–1.28)	0.2812	1.08 (0.92–1.28)	0.3466
CFS (1–3)				
CFS 4	0.83 (0.66–1.03)	0.0960	0.89 (0.71–1.12)	0.3103
CFS 5–6	0.71 (0.59–0.85)	< 0.001	0.78 (0.64–0.95)	0.0157
CFS 7–8	0.71 (0.57–0.87)	0.0014	0.79 (0.63–0.99)	0.0380

*Note*: aHR adjusted for eGFR, wave, age, sex, smoking status, CRP, diabetes, CAD, hypertension and CFS

### Subgroup analysis

Subgroup analyses of individuals with Stage 3a-5 kidney disease that were: first wave patients; aged 65–74 and 85–94; female sex; never and current smoker; CRP ≥ 40 mg/dL; no hypertension; no diabetes; CFS 4 and CFS 5–6 were associated with increased mortality (Additional file 1: Fig. 1). 28-day mortality subgroup analysis of those with a Stage 3a-5 kidney disease found that: first wave patients; aged 85–94, female sex; never and current smoker; CRP ≥ 40 mg/dL; no hypertension; no diabetes; coronary artery disease; and CFS 4 were associated with increased mortality (Additional file 1: Fig. 2). On subgroup analysis of those with Stage 3a-5 kidney disease, no patient characteristics were associated with length of stay in hospital (Additional file 1: Fig. 3).

## Discussion

Our study included 1802 older patients during waves one and two in Europe and we found 41.1% of them died by day 28. Within both the time-to-mortality and 28-day analysis we found a suspected dose-response effect of eGFR, between CKD Stages 1 & 2 and 3b, 4 and 5 with increasing effect, and this appears to be the first study to report this finding within this cohort. Whilst the majority of previous studies have shown the relationship between eGFR and mortality using a binary comparison comparing Stages 1 & 2 versus Stages 3a, 3b, 4, and 5 combined [12, 28], and few have assessed the association comparing groups 3a, 3b, 4, and 5 separately allowing us to assess the likely dose response. Previous work by the Geriatric Medicine Research Collaborative [13] included 5711 individuals and investigated the effect of eGFR categorised in each stage and only found an association in Stages 4 and 5. Our findings extend this work as we report an association between mortality and Stages 3b, 4 and 5. The recent international multicentre HOPE study (Health Outcome Predictive Evaluation for COVID 19) [12] looked at patients of all ages (mean age 66 years old) [12]. They reported that only 8.5% of patients had documented CKD before admission whereas 35% had evidence of renal dysfunction on admission. The study similarly concluded that estimated renal function on admission, documented as eGFR, acted as an independent prognostic factor for mortality in a suspected dose-response pattern [12].

Whilst we identified a dose-response relationship for time to mortality outcome, the greatest association was with an eGFR < 45 (Stages 3b, 4 and 5). This is in line with NICE guidance [29], that states an eGFR of < 45 is an additional risk factor for development of AKI in COVID-19 patients. The results showed that renal function was as strong a predictor of mortality as other key risk factors, such as frailty and age. Therefore, eGFR at < 45 on admission needs to be considered with clinical relevance where identified.

Additionally, we found no association between renal failure and length of stay which was consistent with those from Geriatric Medicine Research Collaborative [13] as neither study report an effect, possibly due to confounding from early mortality.

A number of potential mechanisms of kidney damage in COVID-19 have been hypothesised, including direct viral infection of the kidney via expression of ACE2 receptors in renal cells allowing virus entry which could lead to acute tubular injury and endothelial damage [30]. Damage secondary to cytokine mediated hyperinflammation and thrombotic microangiopathy [31, 32] and systemic illness including sepsis and hypovolaemia [4, 5] has also been described. Age and hypertension specifically, have been associated with increased renal dysfunction and susceptibility to AKI in Covid-19 [11]. Chronic renal impairment is also associated with increased RAAS activity and ACE2 receptors which could also predispose to easier Covid-19 direct cell infection in the kidney.

Our findings should be interpreted in the light of a number of limitations. First, we did not account for underlying renal disease in the patient cohort, therefore renal function calculated on admission did not stipulate between an eGFR due to acute deterioration or chronic renal impairment. Second, this study used a single measurement of kidney function, and did not collect data on longer term kidney function. We also were not able to account for the varying permutations of medications that patients were exposed to. However, so far, this is the largest study to explore the effect of renal impairment in older adults across waves 1 and 2 of the Pandemic, reporting a biologically plausible dose-response.

Our study findings offer important clinical implications, since COVID-19 is anticipated to be embedded as an endemic disease, with new variants circulating globally [33]. Older adults are generally susceptible to COVID-19 and our results improve the identification of older patients with COVID-19 at risk of deterioration, to allow earlier review of risk factors and interventions aimed at preserving and correcting renal dysfunction where possible. Early recognition of renal impairment in older people should inform assessments of prognosis and, where appropriate, inform care escalation decisions. The clear association with deterioration of renal function and increasing age, represents both physiological changes, and also the effect of increased incidence of comorbidity; particularly hypertension, vascular disease and diabetes [34]. In addition, the presence of chronic renal impairment can lead to increased susceptibility to infection [35]. It should be highlighted that older patients may not be suitable for more invasive medical management, including critical care and renal replacement therapy. Therefore, it is even more pertinent that supportive measures are instituted at the earliest opportunity in at risk older patients to prevent further decline. We have identified eGFR may offer an improved prognostic indicator and at seemingly modest decline in renal function for this vulnerable patient cohort.

There are implications of our findings on future research. It is important to better understand the longer-term impact of COVID-19 in those with reduced renal function in survivors, and whether there are both immediate and longer term impacts on clinical outcomes in patients who survive. Further understanding of the impact of renal decline should also be assessed with other clinically important outcomes, such as quality of life, which requires further evaluation. Future research is needed into interventions to improve deranged renal function in older adults.

## Conclusion

Point of care renal failure during admission to hospital, measured by eGFR is a helpful independent predictor of mortality in older patients admitted to hospital with COVID-19. Importance should be placed on either a suspected dose-response, or the clinical implications of increased management may be triggered by Stage 3b renal failure.

## Supplementary Information

**Additional file 1.**

## Data Availability

Due to the confidential nature of the data used within this study, the data is not available for secondary data analysis.

## Abbreviations

aHR: Adjusted hazard ratio
aOR: Adjusted odds ratio
AKI: Acute kidney injury
CFS: Clinical frailty scale
CI: 95% confidence interval
CRP: C-reactive protein
CKD: Chronic kidney disease
CKD-EPI: Chronic Kidney Disease, Creatinine Equation
COPE: COVID-19 in older people study
eGFR: Estimated glomerular filtration rate
HR: Hazard ratio
HRA: Health research authority
IQR: Interquartile ranger
OR: Odds ratio
