# Supplementary material for: Prognostic value of estimated glomerular filtration rate in hospitalised older patients (over 65) with COVID-19: a multicentre, European, observational cohort study
Source: BMC Geriatr. 2022 Feb 12;22:119. doi: 10.1186/s12877-022-02782-5 (PMC8840680; doi:10.1186/s12877-022-02782-5)

Additional Figure 1 – Subgroup analyses of admission eGFR on time to mortality


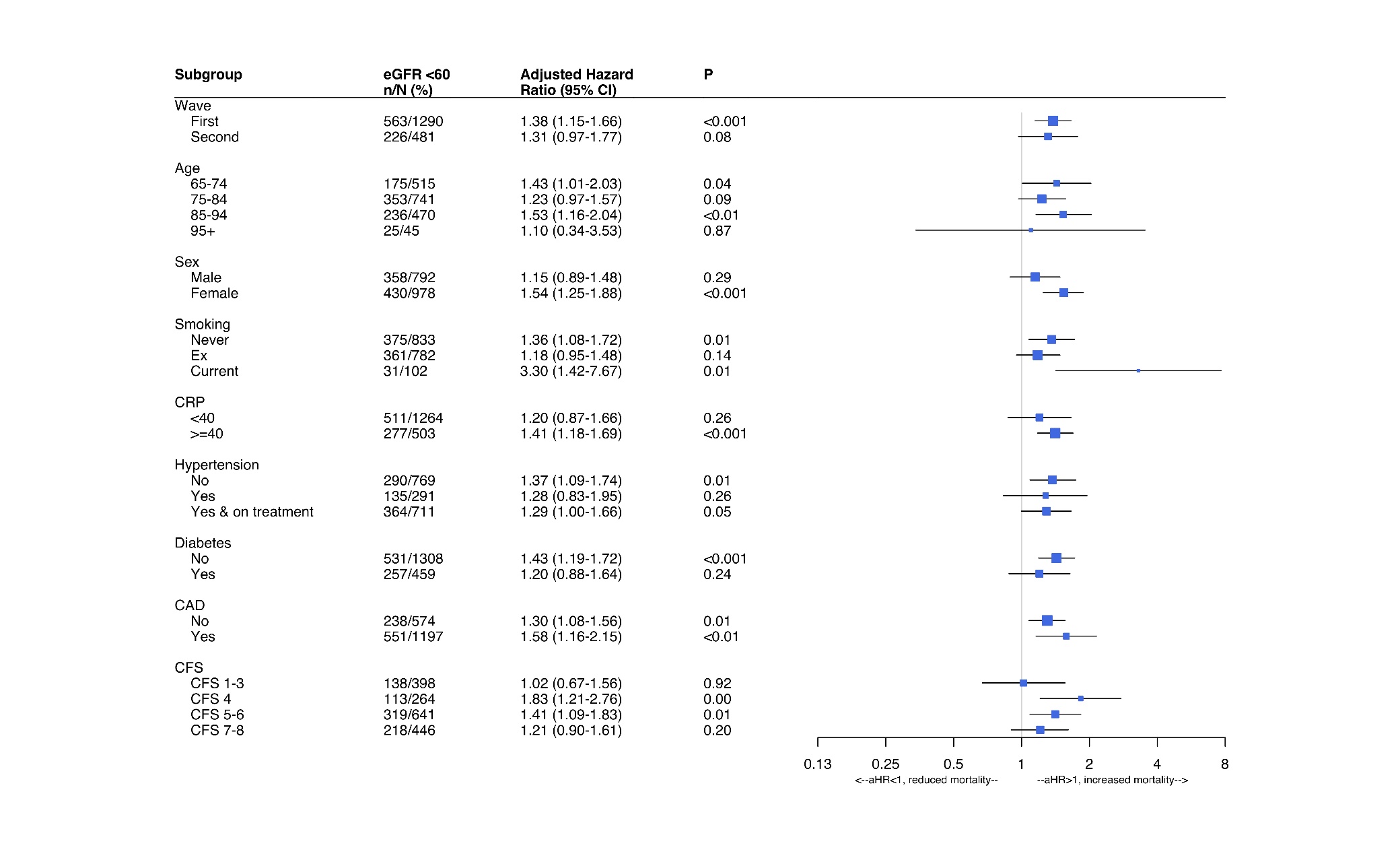


Additional Figure 2 – Subgroup analyses of admission eGFR on Day 28 Mortality


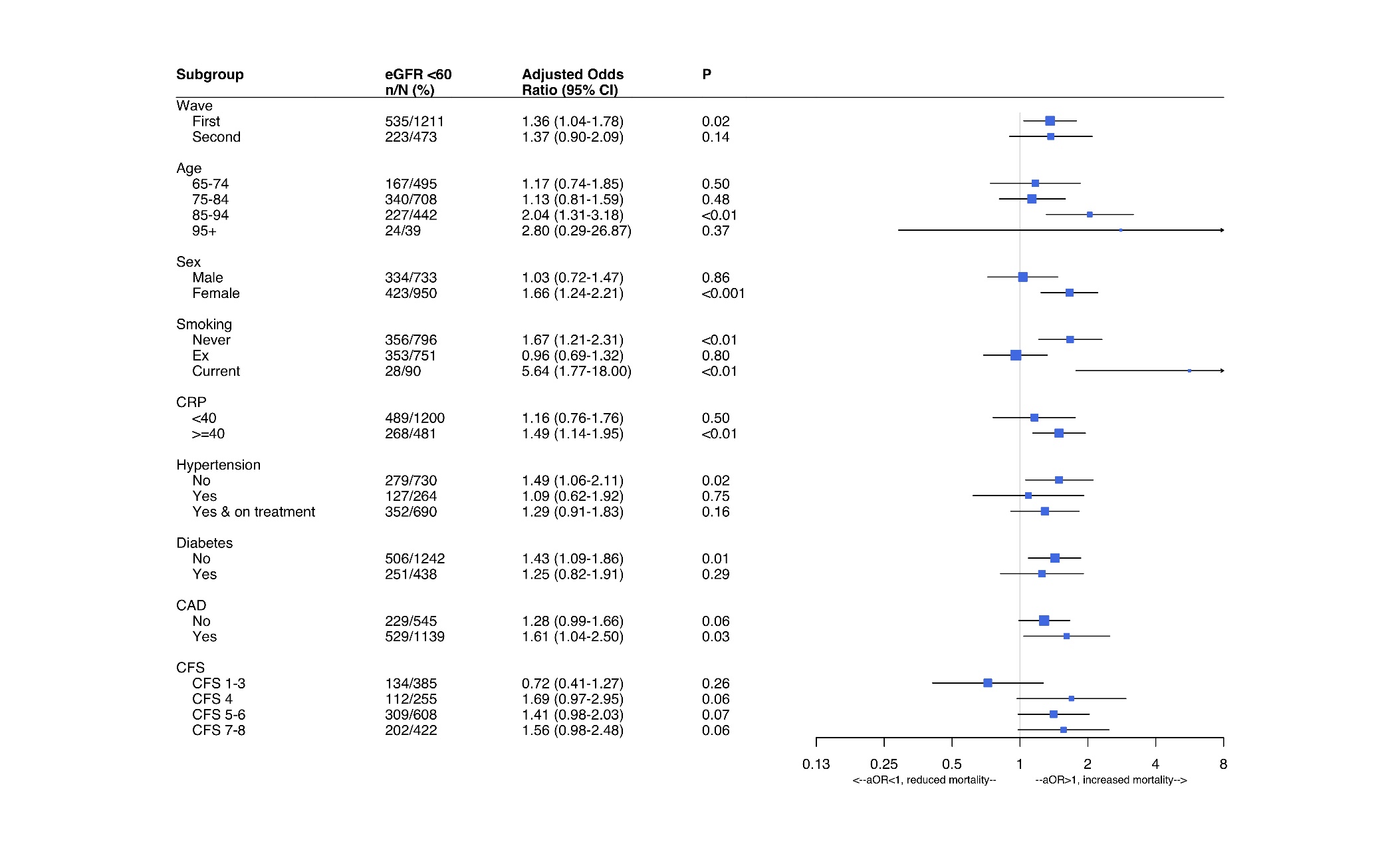


Additional Figure 3 – Subgroup analyses of admission eGFR on length of stay


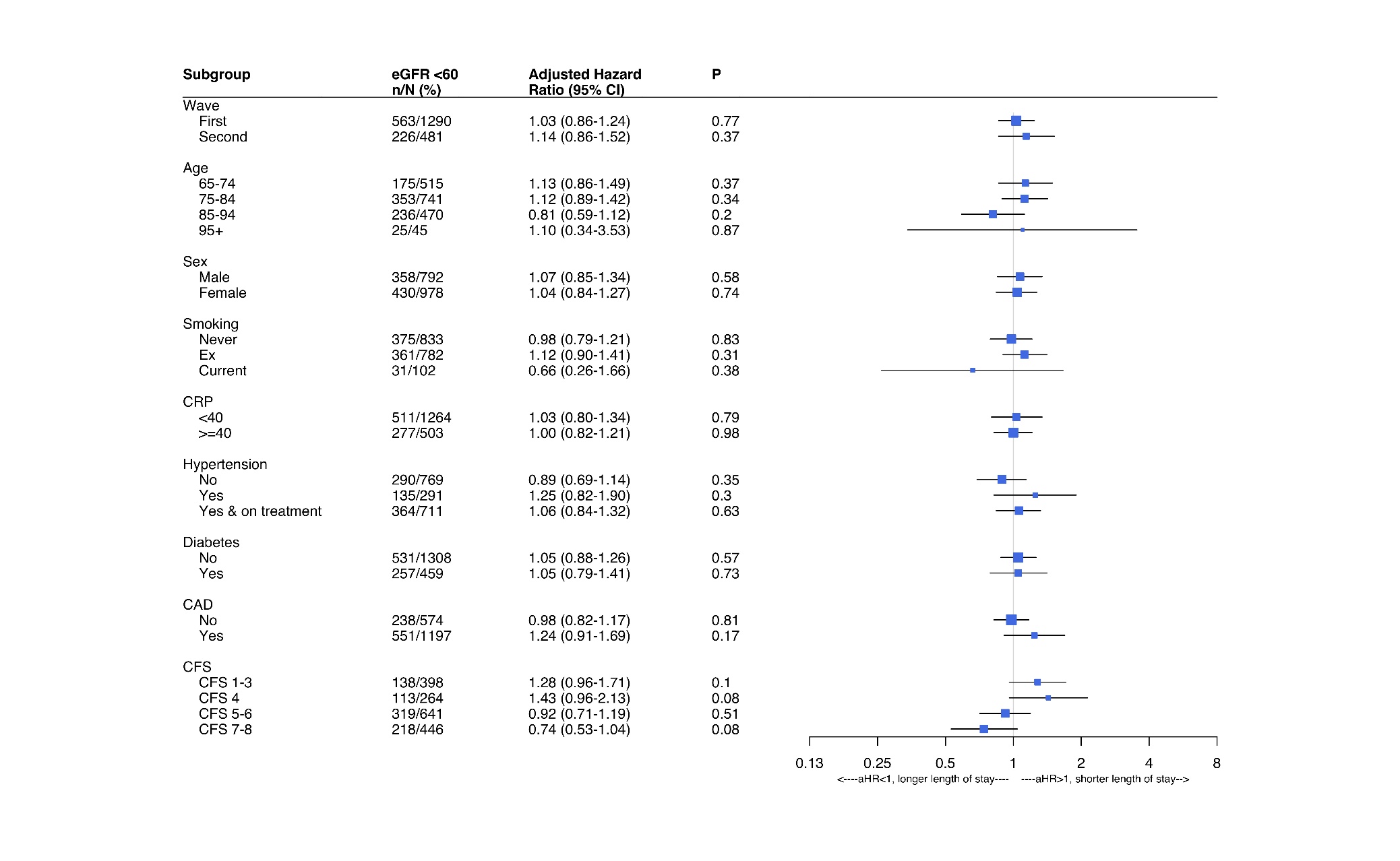

Supplement: Supplementary file 1 — Additional file 1. [file 12877_2022_2782_MOESM1_ESM.docx]
